# A Buffalonian at the West

**Published:** 1857-03

**Authors:** 


### EDITORIAL DEPARTMENT.

---

*A Buffalonian at the West.*—The following letter is from a subscriber recently gone west:

DUBUQUE, January 28, 1857.

*Dear Sir:*

Since my arrival in this western city, I know of no one thing in the way of professional matters, that has given me more pleasure than the perusal of your valuable Journal, and in testimony whereof please receive herewith two dollars for the current year's subscription. Please inform me when my last year's subscription terminated. I have a pleasant office in the business portion of the town, and as I seat myself at the table, and am greeted by the manly and noble countenance portrayed so truthfully in the lithograph of our esteemed friend, and your worthy colleague and predecessor, my memory reverts with no small degree of satisfaction and pleasure to the few short years I spent among you; and then when I take up the familiar friend, the Buffalo Medical Journal, and see A. I. Matthews' annual catalogue of drugs lying before me, I, for a moment, almost forget that I have left you a thousand miles behind, and have become an inhabitant of the far west. But I suppose you will say, enough of this for the present, give us something on the place and climate, medical matters, &c.

Well, the city of Dubuque is situated on the west bank of the Mississippi (here about three-fourths of a mile broad,) and covers the low grounds back to the bluffs, which, at a distance of one-fourth to one-half a mile from the shore, rise almost perpendicularly some one and two hundred feet, and are dotted here and there with fine mansions, embowered in beautiful groves of natural shrubbery. These bluffs are accessible from the lower and business portion of the town, by broad avenues, winding through natural ravines, and although it is now very cold here, and the snow lying in great depth, no one can but admire the native beauties of the scenery, and feel convinced that this is a most picturesque and lovely spot.

The medical profession, I find, has not been entirely forgotten here, for a society has for some time existed here, called the North West Medical Society, comprising within its limits this State, and all the North West; but it now is confined to this county only, and the name has recently been changed to the Dubuque Medical Society. There are some twenty members, (mostly in the city,) and we have regular weekly meetings, which are quite interesting. The officers for the ensuing year are:

| | |
|---|---|
| *President*, - - | Dr. Tom O. Edwards. |
| *Vice-presidents*, - - | " N. B. Mathews & W. W. Woolsey. |
| *Corres. Secretary*, - | " G. Minges. |
| *Recording Secretary*, - | " Benj. McCluer. |
| *Treasurer*, - - | " Wm. Watson. |

*Censors*—Drs. Belden, Horr and Edwards.

*Delegates to Nat. Med. Association*—Drs. Horr, Watson and Finlay.

Our annual meeting took place on the second Tuesday of this month, at which the officers were elected for the year, and other important business transacted, after which, at 9, P. M., the members, with the mayor of the city and a few invited guests, discussed the merits of an excellent supper at the Adams' House.

The cold has been very intense here this winter, the thermometer indicating from 10° to 15° above, to 28° and 37° below zero. This may seem almost fabulous, yet this low temperature is no uncommon thing here, and even at these extremes, nearly all eastern people concur in saying that they do not suffer any more, if as much, as they formerly did in New York State, on the borders of those lakes. There is something very invigorating in the atmosphere, and from its perfect freedom from moisture, perhaps we may attribute the fact that we seldom take cold. It is a well known fact, that many persons of delicate health and weak constitution, those predisposed to pulmonary complaints, have felt less inconvenience from the cold, and have almost invariably been much benefitted by a residence here of a few months. It is not an uncommon thing to see people from Virginia, North and South Carolina, Alabama, Georgia and other southern states, here spending the winter for their health, and some have certainly improved beyond all expectations.

Judging from my own limited experience, and what I have learned from others, bilious diseases and such as are produced by malaria, are those most prevalent here, and seeing in your last number the valuable report on intermittents, by Dr. Nichols, brings to mind that the facts elicited from members of our society who have lived long in malarious districts, go to establish the efficacy of the non-preparatory treatment. Many of the older residents in this western climate, prescribe a dose of eight or ten grains of quinine to be taken just preceding the next anticipated chill. This, they say, almost invariably arrests the disease, and they are very seldom called on to prescribe for a recurrent attack. Some give one or two extra powders to be taken as a precaution on the seventh or twenty-first day after the first powder; but I certainly was surprised to hear that this summary proceeding should so effectually cut short the disease. But I am now trespassing on your time and patience, and will now close. I did not intend this for publication, but if any facts of interest to the profession or the readers of your Journal can be gleaned from this hasty scrawl, you are at liberty to use them as you think best.

I remain,

Yours very respectfully,

James C. Lay.

Dr. Sanford B. Hunt,
Ed. Buffalo Med. Journal.

